# Inter-Organizational Alignment and Implementation Outcomes in Integrated Mental Healthcare for Children and Adolescents: A Cross-Sectional Observational Study

**DOI:** 10.21203/rs.3.rs-3745830/v1

**Published:** 2024-01-02

**Authors:** Yanchen Zhang, Madeline Larson, Mark G. Ehrhart, Kevin King, Aaron R. Lyon, Jill Locke, Clayton Cook

**Affiliations:** University of Washington College of Education; University of Minnesota Twin Cities College of Education and Human Development; University of Central Florida; University of Washington; University of Washington Seattle Campus: University of Washington; University of Washington Seattle Campus: University of Washington; University of Minnesota Twin Cities College of Education and Human Development

**Keywords:** inter-organizational alignment, integrated mental healthcare, organizational implementation context, implementation context, general organizational context

## Abstract

**Background:**

Integrated care involves care provided by a team of professionals, often in non-traditional settings. A common example worldwide is integrated school-based mental health (SBMH), which involves externally employed clinicians providing care at schools. Integrated mental healthcare can improve the accessibility and efficiency of evidence-based practices (EBPs) for vulnerable populations suffering from fragmented traditional care. However, integration can complicate EBP implementation due to overlapping organizational contexts, diminishing the public health impact. Emerging literature suggests that EBP implementation may benefit from the similarities in the implementation context factors between the different organizations in integrated care, which we termed *inter-organizational alignment* (IOA). This study quantitatively explored whether and how IOAs in general and implementation context factors are associated with implementation outcomes in integrated SBMH.

**Methods:**

SBMH clinicians from community-based organizations (CBOs; *n*clinician=27) and their proximal student-support school staff (*n*school=99) rated their schools and CBOs (clinician only) regarding general (*organizational culture and molar climate*) and implementation context factors (*Implementation Climate and Leadership*), and nine common implementation outcomes (e.g., *treatment integrity, service access, acceptability*). The levels of IOA were estimated by intra-class correlations (ICCs). We fitted multilevel models to estimate the standalone effects of context factors from CBOs and schools on implementation outcomes. We also estimated the 2-way interaction effects between CBO and school context factors (i.e., between-setting interdependence) on implementation outcomes.

**Results:**

The IOA in general context factors exceeded those of implementation context factors. The standalone effects of implementation context factors on most implementation outcomes were larger than those of general context factors. Similarly, implementation context factors between CBOs and schools showed larger 2-way interaction effects on implementation outcomes than general context factors.

**Conclusions:**

This study preliminarily supported the importance of IOA in context factors for integrated SBMH. The findings shed light on how IOA in implementation and general context factors may be differentially associated with implementation outcomes across a broad array of integrated mental healthcare settings.

## Introduction

Research has established that fragmented mental health services disproportionately impact the most vulnerable children and adolescents [1, 2, 3]. As a promising solution to increase service accessibility and integration [4], integrated mental healthcare involves a multidisciplinary team of health professionals providing care for clients, often in non-traditional settings (e.g., schools, primary care) [5]. In the US, integrated mental healthcare has gained significant traction [6], partly due to supportive policies (e.g., the Affordable Care Act [7]) and financial investments. Similarly, many countries and regions worldwide have invested in legislation and policies to promote integrated care [8]. Integrated care settings are unique in that they involve overlapping organizational contexts, but little is known about how the two contexts combine and interact to facilitate or impede the uptake and delivery of EBPs.

Implementation research has established that organizational context factors (e.g., general implementation climate) are critical to the development of an enabling and healthy work setting, which impacts individual professionals' EBP implementation outcomes [9, 10, 11]. However, existing research has largely focused on organizational context factors from standalone service settings (e.g., community clinics). Evidence from this siloed approach may not readily transfer to integrated mental healthcare due to its fundamental nature in which interventions are delivered by professionals situated within overlapping contexts (e.g., community-based organizations, CBOs) [12]. To begin to address this knowledge gap, this study aimed to explore and quantitatively illustrate how setting-specific context factors function synergistically (i.e., inter-organizational alignment) to influence implementation outcomes of EBPs in the most common integrated setting for child and adolescent mental health service delivery: school-based mental healthcare.

### Integrated School-Based Mental Healthcare (SBMH)

Schools reduce common barriers to mental healthcare for children and adolescents (particularly those from disadvantaged, ethnic and socioeconomic minoritized groups), which are commonly experienced in traditional outpatient settings [13]. In the US and globally, SBMH services witnessed fast growth, with 50 to 80% of all mental healthcare for children and adolescents now provided in schools [14]. The most common arrangement for SBMH in the US is integrated or co-located SBMH, where services are provided by professionals who are located at school but trained and employed by CBOs external to the education system [15]. Integrated SBMH provides several advantages over traditional outpatient care. First, co-location can minimize service fragmentation by reducing duplicated efforts and enhancing professionals' responsiveness to the needs of children and adolescents [15, 16, 17]. Second, co-locating professionals and their proximal school staff in the same building can enhance their collaboration, shared decision-making, and service integration [13]. Given its public health utility and social significance, integrated SBMH is supported by various policies in the US and internationally [19]. However, EBP implementation in integrated SBMH has been highly variable and inconsistent, which undermines its public health impact [20, 21]. Research examining factors that influence EBP implementation in integrated SBMH is critical to address this gap.

### Implementation Context Factors Relevant to Integrated SBMH

Existing implementation frameworks and models have identified myriad factors that either facilitate or impede EBP implementation in various service settings. While these implementation context factors exist across all levels of an implementation ecology, research has consistently established that context factors from the inner setting of an organization are crucial for the adoption and high-fidelity delivery of EBP [22, 23, 24, 25, 26, 27, 28]. We used the Exploration, Preparation, Implementation, Sustainment (EPIS) framework [2] to identify context factors relevant to integrated SBMH. For instance, general organizational factors, such as organizational culture (shared values, beliefs, and implicit norms that influence staff's behavior) and climate (shared experiences and appraisals of the work environment), are predictive of adoption and use of EBPs [29, 30, 31, 32]. Emerging research has also shed light on the additive effects of implementation organizational factors on staff's implementation behaviors and outcomes. These include implementation climate (shared perceptions of the extent to which implementing EBPs is expected, supported, and rewarded by their organization) and implementation leadership (the attributes and behaviors of leaders that support effective implementation) [33, 34].

Extant implementation literature has examined and consistently endorsed the impacts of context factors on EBP implementation in a single organization or service setting. However, the findings of studies focusing on siloed organizations may not transfer properly to integrated settings such as SBMH. This is partly due to the fundamental nature of co-location that entails embedding professionals from external CBOs into school settings, which is distinct from traditional care where services are provided by professionals located in disparate settings [21]. Hence, research is needed to extend from siloed settings to simultaneously evaluate context factors from different organizations in integrated SBMH. The findings from this integrated approach are instrumental to our understanding of the interactive context factors for successful EBP implementation and the selection or design of corresponding implementation strategies for service quality improvement in integrated care.

### Inter-Organizational Alignment in Integrated SBMH

Based on EPIS and related research, we conceptualize implementation-related *inter-organizational alignment* (IOA) as the degree of similarity in implementation context factors between different organizations involved in integrated care. For instance, organizational-level IOA in implementation climate represents the degree to which staff from different organizations in integrated care settings share similar expectations and experiences of EBP implementation. Prior research in single healthcare organizations has established that intra-organizational alignment (i.e., consistency within a standalone organization) in organizational communication can reduce staff confusion and facilitate their internalization of the priorities and goals of the organization [36, 37, 38, 39, 40]. Thus, we hypothesized that IOA in implementation climate across multiple integrated healthcare organizations would show a similar effect of the intra-organizational alignment in standalone organizations on professionals' implementation behaviors for EBP delivery. To date, there are only qualitative studies that support the importance of alignment in implementation context factors for EBP adoption in inter-organizational collaboration [41, 42, 43]. However, the synergistic effects (i.e., IOA) of context factors from different organizations in integrated care settings have not been examined quantitatively.

**Figure 1:**
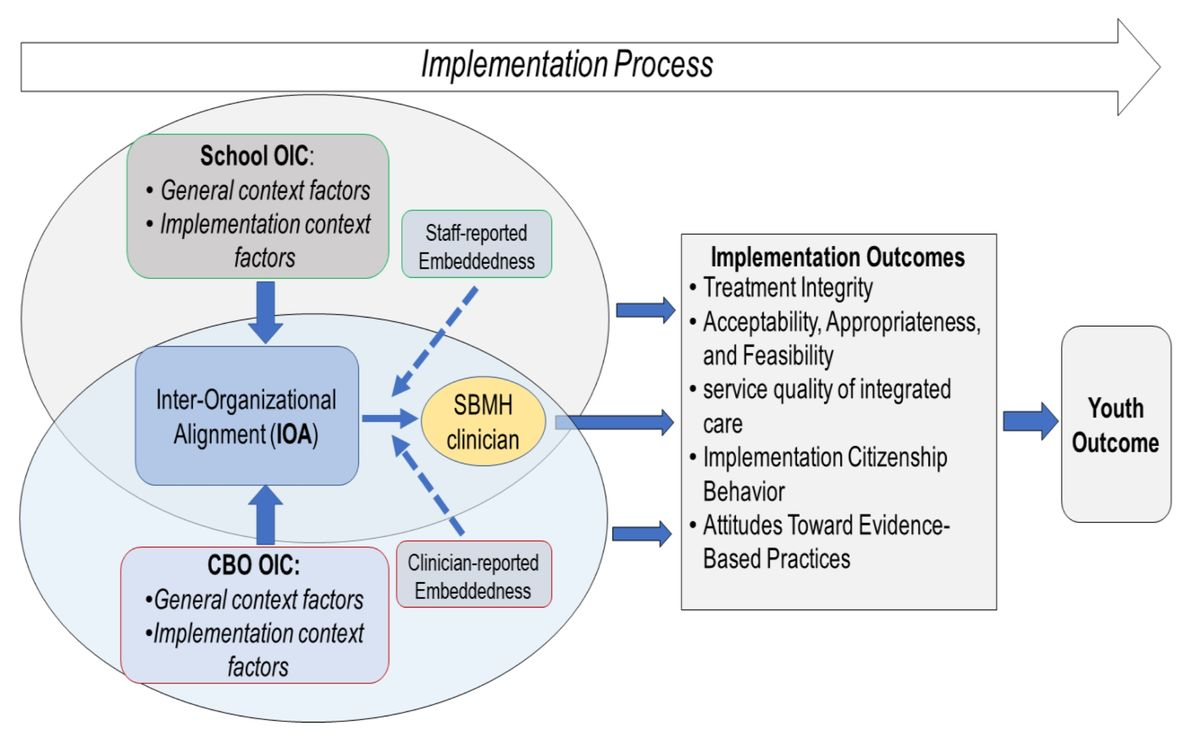
Inter-organizational alignment in organizational implementation context (OIC) in integrated mental healthcare for children and adolescents.

The unique characteristics of integrated SBMH (e.g., co-located care, widely available in the public sector, dual/overlapping administrative relationships between organizations) make it an ideal setting for quantitatively investigating the effects of IOA on EBP implementation [44]. Figure 1 is our conceptualization of the inter-organizational contexts in integrated SBMH. Most integrated SBMH services are delivered by clinicians who are located at school but trained and employed by CBOs external to the education system [45]. This leads to potential discrepancies in the administration and context factors between schools versus CBOs (e.g., training, funding) that influence EBP implementation [46]. Moreover, research has suggested that CBO-employed clinicians are influenced simultaneously by both the school and CBO organizational contexts [45]. Other research has shown that school-based context factors can predict EBP implementation, while implementation outcomes may be contingent on organizational contexts from both CBO and school [47]. In sum, integrated SBMH represents an ideal setting to explore the hypothetical interactive effects on implementation outcomes between context factors from different organizations involved in integrated care (i.e., CBO and school) [48, 49]. Based on existing literature, we hypothesized a positive interaction effect wherein EBP implementation outcomes in integrated SBMH would be highest when context factors in CBO and school are both high.

### Study Aims

Improving the accessibility and effectiveness of EBPs in integrated care requires a fine-grained understanding of how the alignment in context factors between different organizations (i.e., IOAs) are associated with the outcomes of implementation and clients. Despite the promising theoretical propositions from a few qualitative studies [49], no quantitative study exists yet to illustrate the association between IOA and EBP implementation in integrated care. In this cross-sectional observational study, we aimed to explore how IOA between CBO and school context factors is associated with common implementation outcomes in integrated SBMH. This study followed the pre-registered study procedure and analyses published as a study protocol article [66]. To enhance the conciseness and clarity in reporting, we located the content of the ancillary research question (RQ) about clinicians' embeddedness in Additional File 1. Three sequential RQs guided this study.

1. Based on measures reported by clinicians and/or proximal school staff, what are the levels of IOA in implementation context factors between CBOs and schools (general organizational culture and climate, implementation leadership and climate)?
2. What are the standalone main effects of school-versus CBO-based context factors on common implementation outcomes in integrated SBMH (e.g., treatment integrity, improved access, feasibility)?
3. Is the interaction between school- and CBO-based context factors (i.e., IOA) associated with common implementation outcomes in integrated SBMH?

## Methods

### Participants and Settings

Participants were CBO-employed SBMH clinicians and their proximally related school staff (e.g., school nurses, counselors, social workers, or administrators who were involved in supporting or facilitating SBMH programming) from two large urban school districts in the Midwest and Pacific Northwest (*n*_school_=27). CBOs were recruited if they had administrative relationships with schools that reflect the common arrangements nationally (i.e., external CBOs providing SBMH service via a district or county contract; *n*_CBO_=9). In the analytic sample, the CBO clinicians (*n*_clinician_=27) were 92.59% female, 11.11% Hispanic/Latinx, 55.56% Caucasian, 3.7% African American, 7.41% Asian, and everyone held a master’s degree. Their proximal school staff (*n*_school_=99) were 85.86% female, 9.09% Hispanic/Latinx, 73.47% Caucasian, 14.29% African American, 2.04% Asian, and 79.38% with a master’s degree.

### Procedures

IRB approval was obtained from the authors' university. We administered a large-scale online survey to CBO-employed SBMH clinicians and their identified proximal school staff about the context factors and implementation outcomes from their respective organizations. Consent were obtained in the initial section of the survey. To identify each clinician's proximal school staff, the study rolled out in three phases: (a) clinicians were recruited to complete the clinician-version survey, (b) during survey completion, clinicians identified proximal staff from their embedded schools who were responsible for supporting SBMH (e.g., school psychologists, school counselors), and (c) these proximal school staff were recruited by email and/or telephone to complete the school-version survey. Based on organizational research [50], we obtained at least three participants per CBO/school to ensure a reliable assessment of the organizational constructs (e.g., implementation context). To improve response rates, we used backup data collection methods (e.g., weekly reminder emails, telephone follow-ups). For analytic integrity, we used listwise deletion for cases with missingness in implementation outcomes or context factors. In the analytic sample, the response rate was 90% for clinicians and 99% for proximal school staff.

## Measures

### Implementation Outcomes

#### Treatment Integrity.

Based on prior organizational research[51,52], the treatment integrity of EBPs was assessed by a 4-item scale rated by SBMH clinicians on a 5-point Likert scale ranging from 0 "Not at all" to 4 "To a Very Great Extent." A higher score indicates better treatment integrity. Each item assesses a specific dimension of the extent to which a clinician implemented EBPs to students as intended, including *Fidelity, Competence, Knowledge, and Adherence*. In this sample, the internal consistency for this scale was high (*α* = .97).

#### Acceptability, Appropriateness, and Feasibility (AAF).

The AAF of generic EBPs delivered by clinicians was assessed with the Acceptability of Intervention Measure, Intervention Appropriateness Measure, and Feasibility of Intervention Measure, respectively [53]. All items were rated by SBMH clinicians on a 5-point Likert scale ranging from 1 "Completely Disagree" to 5 "Completely Agree". Per the measures' instructions, some item wordings were tailored to refer to generic EBPs. In this sample, all three measures demonstrated good internal consistencies (Cronbach's α: acceptability = .95, appropriateness = .97, and feasibility = .89).

#### Expanded School Mental Health Collaboration Instrument (ESCI).

The proximal school staff completed three subscales of the ESCI to assess their clinicians' service quality in schools [54]. The three subscale scores were used as separate implementation outcomes specific to integrated SBMH in this study, including (a) *Support for Teachers and Students* (how students and teachers are supported through SBMH programming, eight items), (b) *Increased Mental Health Programming* (five items), and (c) *Improved Access for Students and Families* (three items). All items were rated by proximal school staff on a 4-point Likert scale ranging from 1 "never" to 4 "often". In this sample, the three subscales’ Cronbach's α ranged from .79 to .95.

#### Implementation Citizenship Behavior Scale (ICBS).

The SBMH clinicians and their proximal school staff completed the ICBS to report their implementation citizenship behavior (i.e., the degree to which one goes "above and beyond their duty" to implement EBPs) [55]. The ICBS includes six items loading onto two subscales: "Helping Others" and "Keeping Informed". In this study, the total score of ICBS was used with a Cronbach's α of .95.

#### Attitudes Toward Evidence-Based Practices Scale (EBPAS).

The SBMH clinicians and their proximal school staff completed the school version of EBPAS to report their attitudes toward EBPs [56] The school version of EBPAS was adapted for use with service providers in the education sector. It consists of 16 items loading onto four subscales: *Requirements, Appeal, Openness, and Divergence*. In this study, the total score of EBPAS was used, with a Cronbach's α of .91.

### Explanatory Variables: Organizational Context Factors

The SBMH clinicians completed the same measures about the implementation context in two organizations: their employing CBOs and embedded schools. To control for sequential bias, half of the clinicians were randomized to assess their CBO first, while the other half assessed their schools first.

#### Implementation Leadership Scale (ILS).

The ILS [57] has 12 items rated on a 5-point Likert-Scale (0 = "not at all" to 4 "very great extent"), which load onto four subscales, including *Proactive Leadership, Knowledgeable Leadership, Supportive Leadership, and Perseverant Leadership*. When rating for implementation leadership in CBO, the item wordings were tailored for CBO (e.g., "school" replaced with "agency"). In this sample, the SILS demonstrated excellent internal consistency (school α = .98; CBO α = .96).

#### Implementation Climate Scale (ICS).

The ICS [59] assessed the degree to which a school possesses an implementation climate supportive of translating EBPs into routine practice. The ICS includes 18 items loaded onto six subscales which form a total score: *Focus on EBP, Educational Support for EBP, Recognition for EBP, Rewards for EBP, Selection for EBP, and Selection for Openness*. When rating for CBOs, the item wordings were tailored accordingly (e.g., "school" was replaced by "agency"). All items are scored on a 5-point Likert-scale (0 = "not at all" to 4 "very great extent"). In this sample, the ICS demonstrated good internal consistency (school α = .94; CBO α = .91).

#### Organizational Social Context (OSC).

TThe OSC assesses the general (i.e., molar) organizational culture and climate. Given the focus of this study, we selectively administered the *Proficiency* (15 items) subscale from the *General Organizational Culture Scale*, as well as the *Stress* (20 items) and *Functionality* (15 items) subscales from the *General Organizational Climate Scale*. Items were rated by clinicians on a 5-point Likert scale ranging from 1 "Never" to 5 "Always". When rating the CBO, the item wordings were tailored for CBO (e.g., "school" replaced with "agency"). In this sample, the three subscales demonstrated good internal consistency (α ranging from .71 to .93 for schools and from .75 to .91 for CBOs).

### Covariates

To control for potential confounders, the survey collected demographic information from SBMH clinician and their proximal school staff about their age, gender identity, ethnicity, race, education level, and work experience in their current position (Table 1).

## Analysis

We followed the pre-registered analytic procedure [66]. The dataset used for RQ 1 is configured such that the dyads of CBO and school ratings of a context factor (level-1 units) were nested within clinicians (level-2 units). The magnitude of IOA in CBO and school context factors was quantified by the intra-class correlation coefficient [ICCs (2,1), i.e., 2-way mixed effects, single measurement, absolute agreement], which was estimated with random-intercept-only multilevel models (MLMs) using each context factor as the outcome without predictors. We also ran paired-sample t tests to probe the significance of differences in context factors between CBO and schools. Because the measures of context factors differ in their maximum scores, the ratios of means over maximum scores were computed for each context factor. The ratios enabled us to compare the levels of different types of context factors between schools and CBOs because ICCs cannot indicate the directions of IOA (e.g., high/low in both school and CBO).

**Figure 2:**
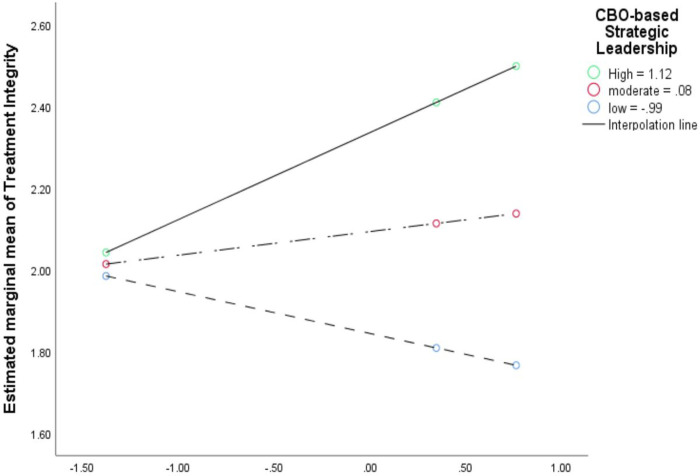
Example of positive/compensatory 2-way interaction effect between CBO versus school context factors (implementation leadership) on implementation outcomes (treatment integrity) in integrated mental healthcare. The predictors (context factors) were group mean centered. Black lines = smoothed regression lines for the three levels of the moderator (CBO-based implementation leadership). Solid line with green dots = high level of moderator (84th percentile), long-dash line with red dots= moderate level of moderator (50th percentile), short-dash lines with blue dots= low level of moderator (16th percentile).

**Figure 3:**
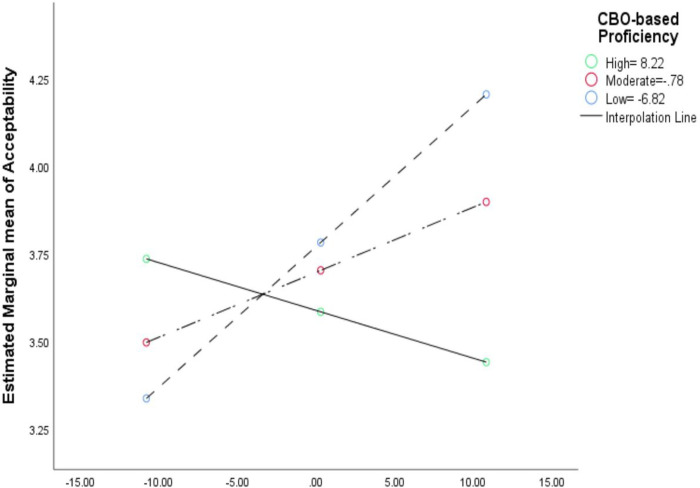
Example of the negative/suppressive 2-way interaction effect between CBO versus school context factors (general factor of Proficiency) on implementation outcomes (perceived acceptability) in integrated mental healthcare. The predictors (context factors) were group mean centered. Black lines = smoothed regression lines for the three levels of the moderator (CBO-based Proficiency). Solid line with green dots = high level of moderator (84th percentile), long-dash line with red dots= moderate level of moderator (50th percentile), short-dash lines with blue dots= low level of moderator (16th percentile).

The dataset used for RQs 2 and 3 was configured so that the SBMH clinicians and their reported context factors and implementation outcomes (level-1 units; n_clinician_=27) were nested in CBOs (level-2 units; n_CBO_=9). The school-based context factors were aggregates of all personnel in each school (i.e., clinicians and their proximal school staff: n_staff_=99). We fitted random-intercept-only MLMs to account for the nesting of clinicians within CBOs (Additional file 2). The dyads of clinician-rated context factors in CBO and school were entered into MLMs as level-1 explanatory variables for each of the nine implementation outcomes (see Measures). Context factors were centered around their group means to adjust for their moderate level of multicollinearity and to enhance the interpretability of their coefficients [63]. In the MLMs, participant demographics did not account for significant portions of variance in the implementation outcomes. Hence, we excluded them from the final models. For RQ 3, we entered 2-way interaction terms between CBO and school context factors to the MLMs in RQ 2. The two-way interaction models allowed us to examine RQ3 and our hypothesis that EBP implementation outcomes in integrated SBMH would be highest when context factors in CBO and school are both high. To facilitate readers to interpret the interaction effects, we plotted two exemplary interactions (positive and negative; Figures 2 and 3, respectively).

Based on the published study protocol, our effect size estimates were expected to resemble the population-level estimates because our sampling frame approximated the SBMH clinician population in the two participating regions [66]. Hence, we focused on interpreting the effect sizes of context factors, instead of statistical significance, to inform practice and future studies (Table 3). We estimated partial Cohen's *d* of all fixed effects to compare across explanatory variables, interaction terms, and models [63]. To complement standardized effect sizes, unstandardized fixed effect coefficients were computed with the empirical Bayes method as generalizable effect estimates [64]. Given the multiple hypothesis tests, p-values would likely produce inflated Type I error. Among the MLMs for each implementation outcome, false discovery rate-corrected *p*-values (i.e., *q*-values) were calculated using the Benjamini-Hochberg method to control for potential false positives with a level of significance of .05 [65]. Analyses were performed with SPSS version 26 and HLM version 6.08. For precision and informativeness for future studies, three decimal points were reported for key statistics. We followed the STROBE checklist for result reporting (Additional file 3).

## Results

### RQ 1: Levels of Inter-Organizational Alignments

We checked basic assumptions (e.g., significant correlations among key variables; Table 2) and confirmed the sample adequacy for MLM. ICCs were calculated as the estimates of the degree of alignment in each implementation contextual factor *between* CBOs and schools (all ICCs reached statistical significance; Table 3). In general, the magnitudes of IOAs were higher in general context factors (ICC=.585 for *Proficiency*; ICC=.282 for *Functionality*; ICC=.831 for Stress) than those in the total scores of *Implementation Climate* (ICC=.342) and *Leadership* (ICC=.167). Between implementation context factors, the average level of IOA among the subscales of Implementation Climate (ICC=.283) exceeded that of Implementation Leadership (ICC=.174; for detailed IOAs of all subscales, see Table 3). Among the subscales of *Implementation Climate, Selection for openness* (ICC=.469) and Focus on *EBP* (ICC=.390) showed the highest levels of IOA, while *Educational support for EBP* showed the lowest level (ICC=.016). Among the subscales of *Implementation leadership, Proactive Leadership* (ICC=.394) showed the highest level of IOA while *Perseverant Leadership* showed the lowest (ICC=.030).

The ICCs imply that the context factors did not perfectly align between CBOs and schools. Hence, we used *t* tests to probe the significance of the between-setting mean difference in context factors. The results indicated that, in general, most context factors in CBOs were higher than those in schools with some of the mean differences reaching statistical significance (e.g., *Implementation Climate* and *Leadership, Functionality, Stress*; Table 3). We compared the ratios of mean over the maximum score for each context factor between schools and CBOs because ICCs cannot indicate whether a context factor is simultaneously high or low in both settings. On average, the absolute level of general context factors exceeded that of Implementation leadership, followed by Implementation *Climate*. Moreover, the absolute levels of *Stress* as well as the total and subscale scores of *Implementation Leadership* in schools exceeded those in CBOs, while the opposite was true for most subscales of *Implementation Climate, Proficiency, and Functionality*.

### Multilevel Models

Tables 4, 5, 6, 7, 8, 9, 10, 11, and 12 summarize the fixed effect sizes of implementation context factors and their interaction terms. Given the large number of results, we focused on the levels of IOA in various context factors, as well as clinically meaningful patterns in effect sizes. We created visualizations (Additional file 4) to compare the effect size and directions of the CBO versus school context factors in a systematic order based on RQs, types of context factors (i.e., general vs. implementation), and implementation outcomes. The results of the standalone main effect MLMs were likely more robust and better powered than the interaction MLMs with more complex configurations.

### RQ 2: Standalone Main Effect MLMs

Several patterns surfaced from the main effects of setting-specific context factors on implementation outcomes. In both CBOs and schools, the effect sizes of *Implementation Climate* and *Leadership* were larger than those of the general context factors (*Proficiency, Stress*, and *Functionality*) on most implementation outcomes. Also, the effect sizes of *Implementation Climate* exceeded those of *Implementation Leadership* for most implementation outcomes, except for Feasibility and *Attitudes toward EBPs* (Tables 10 and 11). Between settings, the effect sizes of implementation and general context factors in CBOs were larger than those in schools for most implementation outcomes, except for *Acceptability, Feasibility, and Attitudes toward EBPs* (Tables 8, 10, and 11). Moreover, the effect directions of implementation context factors in CBOs (more often positive, e.g., Figure 2) were opposite to those in schools (more mixed; e.g., Figure 3) in most implementation outcomes, except for *Support for Teachers and Students and Feasibility* (Tables 5 and 10). Similarly, the effect directions of general context factors in CBOs were opposite against those in schools for most implementation outcomes.

### RQ 3: 2-way Interaction Effects

Although there was no statistical significance due to the limited power, three patterns of 2-way interaction effects were identified based on the types of context factors and implementation outcomes. First, unlike the findings from RQ2, the magnitudes of 2-way interaction effects between CBO- and school-based general context factors were higher than those of implementation context factors on most implementation outcomes, except for *Treatment integrity* and *Implementation citizenship behaviors* (Tables 4 and 12). Second, for *Appropriateness* and *Feasibility* (Tables 9 and 10), the magnitudes of interaction effects between CBO and school *Implementation Leadership* were smaller than those of *Implementation Climate*. The opposite was observed for other implementation outcomes. Third, inconsistent directions of the interaction effects (positive vs. negative) existed between CBO and school context factors (implementation and general) across implementation outcomes. Noteworthy is that both *ImplementationLeadership* and *Climate* showed negative interaction effects for the three implementation outcomes specific to integrated SBMH (i.e., *Support for Teachers and Students, Increased Mental Health Programming, Improved Access for Students and Families*; Tables 5, 6, 7).

## Discussion

Successful implementation of EBPs in integrated mental healthcare requires synergistic efforts of service providers from different organizations and adequate alignment of the implementation contexts of these organizations (i.e., IOA; 66). To date, little is known about how alignment in implementation context factors between multiple organizations influences EBP implementation in integrated care. This is the first quantitative study to narrow this knowledge gap to inform future investigation and practice about EBP implementation in an integrated mental healthcare setting for children and adolescents (e.g., integrated SBMH). Our findings offered preliminary evidence that (a) supported the importance of IOA between CBOs and schools regarding general and implementation context factors, and (b) shed light on how IOA in general and implementation context factors differentially influence common implementation outcomes in integrated SBMH. These results could serve as an empirical foundation to inform future large-scale studies about cost-effective designs and sample planning (e.g., power analysis, starting values for estimation; 67) to effectively power more nuanced and sophisticated analyses about IOA.

### Levels of IOA in Organizational Context Factors

Our findings revealed some intriguing patterns in the levels of IOA in context factors. The average levels of IOA between schools and CBOs were higher in general context factors than implementation ones. The follow-up t tests indicated that slightly larger discrepancies between CBOs and schools existed in the levels of implementation context factors than those of general factors. This may be attributable to the different nature of the two service settings. For instance, the common priority in schools is not implementing EBPs for students' mental health, and it is common for SBMH clinicians to hold various jobs and roles in schools as compared to CBOs. These differences in organizational priorities and job duties could lead to clinicians’ more mixed experiences of school-based implementation climate, which was reflected in the larger variabilities (i.e., standard deviations; Table 3) in their reported context factors in schools than in CBOs. Conversely, clinicians in most CBOs were aware that their organizations prioritized and valued EBP implementation, which may have led to their consistent experience of CBO-based implementation climate. This contrast amplified the between-organization discrepancy (i.e., low IOA in implementation climate).

On the other hand, general context factors represent common social contexts that are likely more pervasive across CBOs and schools than implementation ones. For instance, stress showed the highest level of IOA in schools and CBOs, which was consistent with the literature on pervasive staff burnout in both settings [68]. Similarly, we found that the absolute levels of general context factors exceeded those of implementation context factors. Taking IOA and absolute level together, the levels of general context factors between CBOs and schools appeared to be both better-aligned and higher than those of implementation context factors. This finding further indicates that, compared to the already well-aligned and adequate general context factors, there is more room and need for improvement in the implementation context factors in both CBOs and schools. In sum, these findings suggested that leaders of integrated SBMH should be implementation in allocating resources (e.g., dedicated funding and staffing, leadership meetings between school and CBO, new organizational communication technology; 77,78, 79) to improve the IOA in and absolute levels of key implementation context factors based on their type (general vs. implementation), level of discrepancy (between individual- vs. organizational-differences) and characteristics of service settings (e.g., schools versus CBOs).

### Main Effects of CBO versus School Context Factors

For most implementation outcomes, the effect sizes for the main effects of implementation context factors (e.g., *Implementation climate*) in both CBOs and schools exceeded that of general factors (e.g., *Proficiency*). This finding corroborates the existing body of research to support that implementation context factors have stronger associations with individual-level behavioral and cognitive implementation outcomes (e.g., treatment integrity, acceptability) than general factors, which holds across service settings in integrated care [69]. It also is a key implication for leaders of integrated SBMH to emphasize and foster a positive implementation context that is conducive to the delivery of EBPs. Furthermore, we found that implementation context factors in CBOs showed stronger associations with implementation outcomes compared to the same factors in schools. This implies that SBMH clinicians' behaviors and cognitions related to EBP implementation (e.g., treatment integrity, attitudes toward EBPs) are potentially influenced more by the implementation context in their CBOs than in schools. For example, clinicians’ *knowledge* about and *competency* in EBPs (two items measuring treatment integrity) were influenced more by their employers (CBO) who provide training, supervision, and salary rather than their physical setting (school) where they provide services. This finding has implications for leaders of CBOs who embed their clinicians in other organizations for integrated care. Specifically, leadership-focused implementation strategies (e.g., Leadership and Organizational Change for Implementation, LOCI; 71) could be used at CBOs to improve their implementation context factors which are more closely related to the implementation outcomes of integrated care than those in the actual service provision setting (e.g., schools).

### Two-way Interactions Between CBO and School Context Factors

Compared to implementation context factors, general factors (e.g., *Stress*) in schools and CBOs demonstrated larger 2-way interaction effects in their associations with implementation outcomes. This implies that the effects of school and CBO general context factors depended on each other when it comes to explaining the variability in common implementation outcomes in integrated care. It is consistent with our earlier finding that the levels of IOA between CBOs and schools were higher in general factors than in implementation ones. Due to their different organizational nature and priorities, low levels of IOA in implementation context factors were observed between CBOs and schools. The low IOA (i.e., a large between-organization discrepancy) in implementation context factors in turn restricted their interaction effects on influencing individuals' implementation behaviors. Leaders of integrated SBMH can leverage this finding by prioritizing and coordinating their efforts to deliberately improve their alignment in implementation contexts between CBOs and schools. For instance, at the exploration stage of integrated care, leaders can build their inter-organizational communication to run a collaborative campaign in their organizations advocating for the significance of and rewards for implementing EBPs [72, 73]. Also, leaders in different organizations can coordinate the use of implementation strategies to improve and align their implementation context factors to achieve adequate IOA.

Across different implementation outcomes, the mixed directions of the 2-way interactions implied two types of interdependences (e.g., Figs. 2 and 3; Additional file 4). The 1st type is the compensatory effect, mostly on clinicians' implementation behaviors (e.g., *treatment integrity, implementation citizenship behaviors*). For instance, the highest levels of *Treatment Integrity* were found when there were high levels of *Implementation Leadership* in both settings (CBOs or schools), which aligned with our hypothesis (Fig. 2). The 2nd type is the suppressive effect on *Acceptability* and the implementation outcomes specific to integrated SBMH (e.g., *Increased Mental Health Programming*). For instance, levels of *Acceptability* were highest when levels of *Proficiency* were high in schools but low in CBOs (Fig. 3). This finding differed from our theoretical hypothesis based on prior literature wherein implementation outcomes in integrated SBMH would be highest when the levels of context factors in both CBO and school are high. The fact that the 2-way interaction effects showed a mix of positive and negative directions implies that the nature of the interdependence of context factors between organizations in integrated care may be inconsistent and nonlinear, which is not in line with theoretical predictions. Hence, future research is called for to replicate this study with a large and nationally representative sample (i.e., for higher precision in estimation).

### Limitations and Future Directions

Several limitations exist in this exploratory study that warrant cautious interpretations of the findings and future research. First, The sample was restricted due to the limited number of integrated SBMH settings available in the participating regions. The models were underpowered by design, so we focused on interpreting effect size estimates instead of making statistical inferences [66]. Given the unique organizational structure in integrated SBMH (e.g., one CBO hosts multiple clinicians each of whom serves a single school), future studies can extend this work by recruiting nationally representative samples of integrated SBMH settings. This will enable inferential statistics and advanced modeling (e.g., response surface analysis, 75) that are generalizable to other regions and integrated care settings.

Second, due to the limited sample size, this study took a univariate approach to model each implementation outcome separately. However, the moderate to significant correlations among the implementation outcomes may lead to misestimated standard errors. Future research with multivariate MLMs (e.g., simultaneously modeling the linear combination of multiple implementation outcomes) may yield more precise effect estimates [76]. Third, we used a cross-sectional design given the exploratory aims of this study. Hence, we can only build explanatory models instead of predictive ones. Future studies should use our findings to design longitudinal studies to predict how changes in IOA in the context factors of multiple organizations can influence subsequent implementation outcomes in integrated care.

## Conclusions

Successful EBP implementation in integrated mental healthcare for children and adolescents requires proper alignment in the implementation contexts between organizations. This study is the first to quantitatively explore and illustrate a nascent construct, IOA, in organizational context factors in integrated mental healthcare that involves providers influenced by different organizations. Our findings shed light on how setting-specific context factors were synergistically associated with key implementation outcomes in integrated mental healthcare for children and adolescents. We hope this study can inform leaders and researchers concerning integrated care about the importance of IOA in the context factors of different organizations and how to select specific context factors for their implementation improvement efforts.

## Figures and Tables

**Table 1:** Demographics of School Staff (n_school_ = 99) and CBO Clinicians (n_clinician_ = 27)

		School Staff		CBO Clinicians	
		*n*	%	*n*	%
Age	25 to 34	38	38.38	9	33.33
	35 to 44	26	26.26	11	40.74
	45 to 54	20	20.20	5	18.52
	55 to 64	15	15.15	1	3.70
	65 to 74	-	-	1	3.70
Female	Male	13	13.13	2	7.41
	Female	85	85.86	25	92.59
	Other	1	1.01	-	-
Education	Bachelor’s Degree	15	15.46	-	-
	Master’s Degree	77	79.38	27	100
	Doctoral Degree	5	5.15	-	-
Ethnicity	No	90	90.91	24	88.89
	Yes	9	9.09	3	11.11
Race	American Indian or Alaskan Native	1	1.02	2	7.41
	Asian	2	2.04	4	14.81
	Black or African American	14	14.29	1	3.70
	Native Hawaiian or Other Pacific Islander	2	2.04	-	-
	White or Caucasian	72	73.47	15	55.56
	Other	1	1.02	1	3.70
	Multiracial	6	6.12	4	14.81
Experience in current position	1–5 years	35	35.7	10	37.03
	6–10 years	21	21.42	8	29.63
	11–15 years	12	12.24	4	14.81
	16–19 years	11	11.22	3	11.1
	More than 20 years	19	19.39	2	7.41

**Table 2:** Bi-variate Correlations Among All Variables in the MLMs

	1	2	3	4	5	6	7	8	9	10	11	12	13	14	15	16	17	1
1.ILS_C	26	26	26	26	26	26	26	26	26	26	26	26	26	26	26	26	26	2
2.ILS_S	.28	27	26	27	27	27	27	27	27	27	27	27	27	27	27	27	27	2
3.ICS_C	.59**	.35	26	26	26	26	26	26	26	26	26	26	26	26	26	26	26	2
4.ICS_S	.20	.53**	.39*	27	27	27	27	27	27	27	27	27	27	27	27	27	27	2
5.Prof_C	.61**	.19	.43*	.20	27	27	27	27	27	27	27	27	27	27	27	27	27	2
6.Prof_S	.41*	.22	.30	.25	.61**	27	27	27	27	27	27	27	27	27	27	27	27	2
7.Stress_C	−.02	−.06	.09	.24	−.04	−.08	27	27	27	27	27	27	27	27	27	27	27	2
8.Stress_S	−.06	−.10	−.03	.28	.05	−.04	.83**	27	27	27	27	27	27	27	27	27	27	2
9.Func_C	.45*	.24	.32	.14	.39*	.17	−.12	−.07	27	27	27	27	27	27	27	27	27	2
10. Func_S	.58**	.21	.17	.10	.55**	.45*	−.07	−.12	.38*	27	27	27	27	27	27	27	27	2
11. Embed	0	−.03	−.08	−.09	0	0	−.05	−.08	−.40*	−.18	27	27	27	27	27	27	27	2
12. Integrity	.34	.16	.61**	.16	.10	.05	.05	.04	.05	−.08	−.13	27	27	27	27	27	27	2
13. Support	−.18	.09	−.23	−.11	.01	−.05	−.05	.15	−.03	−.01	.38	−.47*	27	27	27	27	27	2
14.Program	.09	.10	.23	−.02	.07	−.01	−.35	−.25	.07	.16	.41*	−.31	.54**	27	27	27	27	2
15. Access	.25	.02	.31	−.07	.18	−.13	−.01	0	−.05	.17	.36	−.11	.27	.73**	27	27	27	2
16. acceptable	.33	.32	.56**	.28	.05	.19	−.28	−.35	.09	−.01	−.04	.37	−.26	.23	.18	27	27	2
17. appropriate	.36	.41*	.59**	.38	.17	.27	−.31	−.36	.19	.07	−.09	.30	−.33	.20	.19	.93**	27	2
18. Feasible	.17	.32	.4*	.18	−.06	.05	−.37	−.34	.14	−.10	−.12	.36	−.19	.22	.15	.90**	.88**	2
19. EBPAS	.39*	.35	.48*	.10	.05	.13	−.34	−.53**	.09	−.03	−.16	.47*	−.35	−.05	−.07	.78**	.74**	.7
20. ICBS	.52**	.53**	.70**	.51**	.24	.17	.20		.23	.23	−.30	.58**	−.4*	−.20	−.01	.53**	.51**	.3

Note.

* *p* < .05

** *p* < .01

*** *p* < .001.

The number above the diagonal line indicates the sample size used for calculation. All variables were raw, i.e., not centered. “_C” = CBO-based context factor, “_S”=school-based context factor. SILS = School Implementation Leadership Scale, ICS = School Implementation Climate Scale. EPBAS = Attitudes Toward Evidence-Based Practices Scale. ICBS = Implementation Citizenship Behavior Scale. “Prof” = *Proficiency,* “Func” = *Functionality,* embed = *Embeddedness,* Support = *Support for Teachers and Students,* Program = *Increased Mental Health Programming,* Access = *Improved Access for Students and Families*.

**Table 3:** ICCs for All Key Variables of Implementation Context factors

Context Factors	Mean Scores				Ratios of Mean Over Maximum Score Possible		Inter-Organizational Alignment (IOA)
	School	CBO	Mean diff.	t-tests	School	CBOs	School and CBO
S-ICS Total Score (all subscales: max score = 4)	1.24(0.67)	1.59(0.66)	−0.35*	t(25)=−2.41, *p=* .02	.385	.398	.342
Focus on EBP	1.33(0.98)	2.05(1.24)	−0.72**	t(26)=−3.29, *p <* .01	.490	.513	.390
Educational Support for EBP	0.9(0.77)	1.65(1.06)	−0.75**	t(26)=−3.17, *p<* .01	.370	.413	.016
Recognition for EBP	1.09(1.04)	1.56(1)	−0.47*	t(26)=−2.28, *p=* .03	.398	.390	.391
Rewards for EBP	0.57(0.68)	0.36(0.61)	0.21	t(26)=1.44, *p=* .16	.133	.090	.276
Selection for EBP	1.13(0.85)	1.28(1.06)	−0.15	t(25)=−0.59, *p=* .56	.325	.320	.158
Selection for Openness	2.31(1.1)	2.7(0.82)	−0.4*	t(26)=−2.11, *p=* .04	.600	.675	.469
mean IOA across all subscales of ICS							.283
S-ILS Total Score (all subscales: Max score = 4)	1.65(1.02)	1.88(0.97)	−0.75**	t(26)=−3.17, *p<* .01	.538	.470	.167
Proactive Leadership	1.32(0.99)	1.05(0.97)	0.27	t(25)=1.43, *p=* .16	.453	.263	.394
Knowledgeable Leadership	1.6(1.09)	2.18(1.15)	−0.58*	t(25)=−2.28, *p=* .03	.558	.545	.211
Supportive Leadership	1.94(1.18)	2.29(1.09)	−0.36	t(25)=−1.23, *p=* .23	.603	.573	.063
Perseverant Leadership	1.76(1.13)	2(1.2)	−0.24	t(25)=−0.76, *p=* .45	.535	.500	.029
mean IOA across all subscales of ILS							.174
General Context Factors							
Proficiency (Max score = 75)	59.7(10.58)	61.78(7.19)	−2.07	t(26)=−1.28, *p=* .21	.809	.824	.585
Functionality (Max score = 100)	56.78(12.78)	53.1(11.34)	3.68*	t(26)=2.65, *p=* .01	.484	.516	.282
Stress (Max score = 75)	47.81(4.55)	51.59(10.18)	−3.78*	t(26)=−2.08, *p=* .05	.789	.708	.831
mean IOA across all general context factors							.566

Note.

* *p* < .05

** *p* < .01.

ICC (2-way mixed, single measure, absolute agreement) = inter-organizational alignment, Level 1: *N* = 27; Level 2: *N* = 9. S-ICS= School Implementation Climate Scale; S-ILS = School Implementation Leadership Scale, diff. = difference, Standard Deviations were reported in parentheses.

**Table 4:** Models for Treatment Integrity: Fixed Effects of Implementation Context Factors

implementation outcome	Research Questions	Model based on IVs	Fixed effect Coefficients (partial Cohen’s d in parentheses)				
			CBO	School	OAE	IOA (CBO × School)	CBO × School × OAE
Treatment Integrity	Main effects	ILS	0.16 (0.534)	0.047 (0.169)	-	-	-
		ICS	0.489 (0.991)	−0.163 (−0.179)	-	-	-
		OSC1: Proficiency	0.029 (0.5)	−0.019 (−0.555)	-	-	-
		OSC2 Stress	−0.002 (−0.068)	0.01 (0.204)	-	-	-
		OSC3 Functionality	−0.034 (−0.469)	0.052 (0.349)	-	-	-
	2-way interaction	ILS	0.212 (1.328)	−0.079 (−0.365)	-	0.306 (1.454)	-
		ICS	0.482 (1.389)	−0.105 (−0.137)	-	−0.239 (−0.504)	-
		OSC1: Proficiency	0.03 (0.512)	−0.02 (−0.569)	-	0 (−0.072)	-
		OSC2 Stress	0.013 (0.282)	−0.012 (−0.191)	-	−0.002 (−0.519)	-
		OSC3 Functionality	−0.033 (−0.4)	0.049 (0.281)	-	−0.001 (−0.064)	-
	3-way interaction	ILS	0.219 (0.682)	−0.064 (−0.199)	−0.248 (−0.177)	-	1.55 (0.592)
		ICS	0.38 (0.677)	−0.02 (−0.041)	0.353 (0.384)	-	−3.485 (−0.626)
		OSC1: Proficiency	0.029 (0.34)	−0.023 (−0.372)	−0.292 (−0.152)	-	−0.021 (−0.293)
		OSC2 Stress	0.023 (0.35)	−0.011 (−0.147)	0.977 (0.386)	-	−0.019 (−0.799)
		OSC3 Functionality	−0.016 (−0.194)	0.044 (0.317)	−1.171 (−0.659)	-	0.046 (0.605)

*Note.* Level 1: *N* = 27; Level 2: *N* = 9. CBO = Community-Based Organization, ILS = Implementation Leadership Scale, ICS = Implementation Climate Scale, OSC = Organizational Social Context, OAE = *Outreach and Approach* subscale of ESMHC., which measures SBMH clinicians’ embeddedness. For discussion about 3-way interaction with embeddedness, see Additional File 1.

**Table 5:** Models for Support for Teachers and Students: Fixed Effects of Implementation Context Factors

implementation outcome	Research Questions	Model based on IVs	Fixed effect Coefficients (partial Cohen’s d in parentheses)				
			CBO	School	OAE	IOA (CBO × School)	CBO × School × OAE
Support for Teachers and Students	Main effects	ILS	−0.098 (−0.49)	0.063 (0.331)	-	-	-
		ICS	−0.101 (−0.284)	0.026 (0.078)	-	-	-
		OSC1: Proficiency	03 (0.081)	−04 (−0.133)	-	-	-
		OSC2 Stress	−0.018 (−0.625)	0.016 (0.561)	-	-	-
		OSC3 Functionality	01 (0.016)	−0.011 (−0.162)	-	-	-
	2-way interaction	ILS	−0.112 (−0.562)	0.097 (0.472)	-	−0.081 (−0.406)	-
		ICS	−0.102 (−0.286)	0.032 (0.091)	-	−0.025 (−0.054)	-
		OSC1: Proficiency	03 (0.064)	−03 (−0.112)	-	0 (0.093)	-
		OSC2 Stress	−0.026 (−0.876)	0.028 (0.866)	-	01 (0.669)	-
		OSC3 Functionality	01 (0.015)	−0.011 (−0.154)	-	0 (01)	-
	3-way interaction	ILS	−0.08 (−0.477)	0.083 (0.495)	1.117 (1.559)	-	−0.888 (−0.653)
		ICS	−0.136 (−0.453)	−0.044 (−0.158)	0.455 (0.843)	-	−0.614 (−0.264)
		OSC1: Proficiency	−01 (−0.024)	02 (0.082)	0.705 (0.866)	-	06 (0.195)
		OSC2 Stress	−0.016 (−0.588)	0.011 (0.349)	0.686 (0.642)	-	02 (0.182)
		OSC3 Functionality	−01 (−0.034)	02 (0.034)	0.873 (1.149)	-	−09 (−0.288)

*Note.* Level 1: *N* = 27; Level 2: *N* = 9. CBO = Community-Based Organization, ILS = Implementation Leadership Scale, ICS = Implementation Climate Scale, OSC = Organizational Social Context, OAE = *Outreach and Approach* subscale of ESMHC., which measures SBMH clinicians’ embeddedness. For discussion about 3-way interaction with embeddedness, see Additional File 1.

**Table 6:** Models for Increased Mental Health Programming: Fixed Effects of Implementation Context Factors

implementation outcome	Research Questions	Model based on IVs	Fixed effect Coefficients (partial Cohen’s d in parentheses)				
			CBO	School	OAE	IOA (CBO × School)	CBO × School × OAE
Increased Mental Health Programming	Main effects	ILS	0.058 (0.26)	−0.037 (−0.175)	-	-	-
		ICS	0.06 (0.152)	0 (0)	-	-	-
		OSC1: Proficiency	0.027 (0.585)	−0.023 (−0.687)	-	-	-
		OSC2 Stress	−0.018 (−0.505)	07 (0.216)	-	-	-
		OSC3 Functionality	−04 (−0.089)	0.027 (0.336)	-	-	-
	2-way interaction	ILS	0.027 (0.131)	0.037 (0.174)	-	−0.182 (−0.869)	-
		ICS	0.059 (0.151)	04 (0.01)	-	−0.015 (−0.029)	-
		OSC1: Proficiency	0.03 (0.668)	−0.026 (−0.783)	-	−01 (−0.454)	-
		OSC2 Stress	−0.028 (−0.752)	0.022 (0.547)	-	01 (0.62)	-
		OSC3 Functionality	−02 (−0.032)	0.021 (0.25)	-	−02 (−0.244)	-
	3-way interaction	ILS	0.131 (1.022)	0.012 (0.096)	0.647 (1.162)	-	−1.342 (−1.29)
		ICS	0.166 (0.485)	−0.133 (−0.419)	0.465 (0.753)	-	1.208 (0.453)
		OSC1: Proficiency	0.04 (0.965)	−0.017 (−0.563)	0.447 (0.469)	-	−0.013 (−0.357)
		OSC2 Stress	−0.034 (−1.087)	0.019 (0.552)	−0.623 (−0.519)	-	0.015 (1.298)
		OSC3 Functionality	−06 (−0.131)	0.04 (0.56)	0.943 (1.026)	-	−06 (−0.158)

*Note.* Level 1: *N* = 27; Level 2: *N* = 9. CBO = Community-Based Organization, ILS = Implementation Leadership Scale, ICS = Implementation Climate Scale, OSC = Organizational Social Context, OAE = *Outreach and Approach* subscale of ESMHC., which measures SBMH clinicians’ embeddedness. For discussion about 3-way interaction with embeddedness, see Additional File 1.

**Table 7:** Models for Improved Access for Students and Families: Fixed Effects of Implementation Context Factors

implementation outcome	Research Questions	Model based on IVs	Fixed effect Coefficients (partial Cohen’s d in parentheses)				
			CBO	School	OAE	IOA (CBO × School)	CBO × School × OAE
Improved Access for Students and Families	Main effects	ILS	0.104 (0.549)	−0.075 (−0.416)	-	-	-
		ICS	0.168 (0.505)	−0.122 (−0.392)	-	-	-
		OSC1: Proficiency	0.039 (1.23)	−0.03 (−1.303)	-	-	-
		OSC2 Stress	−05 (−0.156)	07 (0.263)	-	-	-
		OSC3 Functionality	−0.034 (−1.021)	0.066 (1.176)	-	-	-
	2-way interaction	ILS	0.073 (0.431)	−01 (−04)	-	−0.181 (−1.064)	-
		ICS	0.163 (0.498)	−0.089 (−0.272)	-	−0.14 (−0.324)	-
		OSC1: Proficiency	0.044 (1.579)	−0.034 (−1.69)	-	−01 (−1.118)	-
		OSC2 Stress	−08 (−0.247)	0.012 (0.361)	-	0 (0.252)	-
		OSC3 Functionality	−0.032 (−0.951)	0.062 (1.063)	-	−01 (−0.227)	-
	3-way interaction	ILS	0.152 (1.212)	02 (0.018)	0.792 (1.474)	-	−0.364 (−0.357)
		ICS	0.318 (1.198)	−0.219 (−0.886)	0.486 (1.016)	-	1.977 (0.956)
		OSC1: Proficiency	0.049 (1.86)	−0.028 (−1.482)	0.391 (0.652)	-	−05 (−0.212)
		OSC2 Stress	−0.014 (−0.492)	0.011 (0.352)	−0.37 (−0.349)	-	0.011 (1.125)
		OSC3 Functionality	−0.032 (−1.116)	0.077 (1.623)	0.584 (0.948)	-	04 (0.135)

*Note.* Level 1: *N* = 27; Level 2: *N* = 9. CBO = Community-Based Organization, ILS = Implementation Leadership Scale, ICS = Implementation Climate Scale, OSC = Organizational Social Context, OAE = *Outreach and Approach* subscale of ESMHC., which measures SBMH clinicians’ embeddedness. For discussion about 3-way interaction with embeddedness, see Additional File 1.

**Table 8:** Models for Acceptability : Fixed Effects of Implementation Context Factors

implementation outcome	Research Questions	Model based on IVs	Fixed effect Coefficients (partial Cohen’s d in parentheses)				
			CBO	School	OAE	IOA (CBO × School)	CBO × School × OAE
Acceptability	Main effects	ILS	0.244 (0.468)	0.139 (0.279)	-	-	-
		ICS	0.062 (0.067)	0.393 (0.448)	-	-	-
		OSC1: Proficiency	0.012 (0.122)	−09 (−0.123)	-	-	-
		OSC2 Stress	−05 (−0.067)	−08 (−0.11)	-	-	-
		OSC3 Functionality	0.012 (0.188)	0.046 (0.423)	-	-	-
	2-way interaction	ILS	0.268 (0.829)	0.08 (0.235)	-	0.144 (0.788)	-
		ICS	0.06 (0.064)	0.411 (0.439)	-	−0.077 (−0.058)	-
		OSC1: Proficiency	0.027 (0.338)	−0.021 (−0.569)	-	−04 (−1.991)	-
		OSC2 Stress	−01 (−0.015)	−0.014 (−0.387)	-	−01 (−0.156)	-
		OSC3 Functionality	0.014 (0.137)	0.043 (0.242)	-	−01 (−0.065)	-
	3-way interaction	ILS	0.316 (0.606)	0.052 (0.099)	−1.784 (−0.785)	-	−0.447 (−0.105)
		ICS	0.748 (0.812)	0.238 (0.296)	−0.101 (−0.066)	-	9.341 (1.059)
		OSC1: Proficiency	0.042 (0.394)	−0.029 (−0.387)	−1.228 (−0.47)	-	02 (0.016)
		OSC2 Stress	−0.022 (−0.219)	09 (0.077)	−1.755 (−0.441)	-	0.012 (0.324)
		OSC3 Functionality	0.054 (0.555)	0.038 (0.233)	−2.122 (−0.997)	-	0.117 (1.238)

*Note.* Level 1: *N* = 27; Level 2: *N* = 9. CBO = Community-Based Organization, ILS = Implementation Leadership Scale, ICS = Implementation Climate Scale, OSC = Organizational Social Context, OAE = *Outreach and Approach* subscale of ESMHC., which measures SBMH clinicians’ embeddedness. For discussion about 3-way interaction with embeddedness, see Additional File 1.

**Table 9:** Models for Appropriateness: Fixed Effects of Implementation Context Factors

implementation outcome	Research Questions	Model based on IVs	Fixed effect Coefficients (partial Cohen’s d in parentheses)				
			CBO	School	OAE	IOA (CBO × School)	CBO × School × OAE
Appropriateness	Main effects	ILS	0.338 (0.716)	0.208 (0.461)	-	-	-
		ICS	0.194 (0.223)	0.517 (0.632)	-	-	-
		OSC1: Proficiency	0.029 (0.292)	−01 (−0.012)	-	-	-
		OSC2: Stress	−01 (−0.013)	−09 (−0.114)	-	-	-
		OSC3: Functionality	0.026 (0.302)	0.055 (0.374)	-	-	-
	2-way interaction	ILS	0.353 (0.741)	0.172 (0.348)	-	0.088 (0.175)	-
		ICS	0.185 (0.214)	0.59 (0.686)	-	−0.305 (−0.249)	-
		OSC1: Proficiency	0.044 (0.454)	−0.014 (−0.197)	-	−04 (−0.806)	-
		OSC2: Stress	08 (0.096)	−0.022 (−0.232)	-	−01 (−0.229)	-
		OSC3: Functionality	0.034 (0.376)	0.038 (0.241)	-	−04 (−0.329)	-
	3-way interaction	ILS	0.429 (0.904)	0.208 (0.437)	−1.733 (−0.838)	-	0.259 (0.067)
		ICS	0.812 (1.016)	0.486 (0.704)	−0.547 (−0.417)	-	9.712 (1.231)
		OSC1: Proficiency	0.075 (0.786)	−0.017 (−0.251)	−1.393 (−0.603)	-	0.01 (0.121)
		OSC2: Stress	−0.025 (−0.262)	09 (0.088)	−2.424 (−0.643)	-	0.022 (0.609)
		OSC3: Functionality	0.07 (0.798)	0.038 (0.261)	−2.011 (−1.052)	-	0.105 (1.219)

*Note.* Level 1: *N* = 27; Level 2: *N* = 9. CBO = Community-Based Organization, ILS = Implementation Leadership Scale, ICS = Implementation Climate Scale, OSC = Organizational Social Context, OAE = *Outreach and Approach* subscale of ESMHC., which measures SBMH clinicians’ embeddedness. For discussion about 3-way interaction with embeddedness, see Additional File 1.

**Table 10:** Models for Feasibility: Fixed Effects of Implementation Context Factors

implementation outcome	Research Questions	Model based on IVs	Fixed effect Coefficients (partial Cohen’s d in parentheses)				
			CBO	School	OAE	IOA (CBO × School)	CBO × School × OAE
Feasibility	Main effects	ILS	0.016 (0.032)	0.241 (0.503)	-	-	-
		ICS	−0.225 (−0.25)	0.236 (0.279)	-	-	-
		OSC1: Proficiency	−05 (−0.053)	−09 (−0.136)	-	-	-
		OSC2 Stress	−0.034 (−0.49)	0.013 (0.198)	-	-	-
		OSC3 Functionality	−01 (−09)	0.039 (0.239)	-	-	-
	2-way interaction	ILS	0.027 (0.052)	0.215 (0.41)	-	0.061 (0.115)	-
		ICS	−0.244 (−0.285)	0.387 (0.452)	-	−0.627 (−0.515)	-
		OSC1: Proficiency	06 (0.065)	−0.019 (−0.275)	-	−03 (−0.62)	-
		OSC2 Stress	−0.034 (−0.433)	0.014 (0.157)	-	0 (05)	-
		OSC3 Functionality	03 (0.032)	0.029 (0.17)	-	−02 (−0.162)	-
	3-way interaction	ILS	0.139 (0.297)	0.255 (0.544)	−2.223 (−1.096)	-	1.023 (0.269)
		ICS	0.189 (0.207)	0.352 (0.442)	−0.511 (−0.336)	-	6.715 (0.763)
		OSC1: Proficiency	0.042 (0.455)	−0.023 (−0.358)	−1.637 (−0.732)	-	09 (0.111)
		OSC2 Stress	−0.051 (−0.603)	0.04 (0.429)	−0.386 (−0.117)	-	−05 (−0.149)
		OSC3 Functionality	0.035 (0.362)	0.037 (0.231)	−2.264 (−1.082)	-	0.107 (1.152)

*Note.* Level 1: *N* = 27; Level 2: *N* = 9. CBO = Community-Based Organization, ILS = Implementation Leadership Scale, ICS = Implementation Climate Scale, OSC = Organizational Social Context, OAE = *Outreach and Approach* subscale of ESMHC., which measures SBMH clinicians’ embeddedness. For discussion about 3-way interaction with embeddedness, see Additional File 1.

**Table 11:** Models for Attitudes about EBP: Fixed Effects of Implementation Context Factors

implementation outcome	Research Questions	Model based on IVs	Fixed effect Coefficients (partial Cohen’s d in parentheses)				
			CBO	School	OAE	IOA (CBO × School)	CBO × School × OAE
Attitudes about EBP	Main effects	ILS	0.083 (0.455)	0.148 (0.926)	-	-	-
		ICS	0.18 (0.408)	−0.037 (−0.062)	-	-	-
		OSC1: Proficiency	0.016 (0.378)	−0.008 (−0.314)	-	-	-
		OSC2 Stress	0.01 (0.709)	−0.025 (−0.976)	-	-	-
		OSC3 Functionality	0.016 (0.285)	0.004 (0.031)	-	-	-
	2-way interaction	ILS	0.123 (1.024)	0.051 (0.182)	-	0.234 (1.156)	-
		ICS	0.186 (0.337)	−0.085 (−0.161)	-	0.197 (0.337)	-
		OSC1 : Proficiency	0.011 (0.246)	−0.004 (−0.155)	-	0.001 (1.538)	-
		OSC2 Stress	0.017 (0.711)	−0.037 (−0.891)	-	−0.001 (−0.499)	-
		OSC3 Functionality	0.011 (0.176)	0.015 (0.101)	-	0.003 (0.402)	-
	3-way interaction	ILS	0.102 (0.453)	0.083 (0.368)	−1.249 (−1.27)	-	0.312 (0.171)
		ICS	0.206 (0.41)	0.033 (0.077)	−0.492 (−0.603)	-	−0.042 (−0.008)
		OSC1: Proficiency	0.022 (0.42)	−0.013 (−0.347)	−1.197 (−0.908)	-	0.01 (0.214)
		OSC2 Stress	0.007 (0.16)	−0.017 (−0.343)	−0.621 (−0.357)	-	−0.004 (−0.236)
		OSC3 Functionality	0.03 (0.598)	0.006 (0.068)	−1.589 (−1.449)	-	0.053 (1.074)

*Note.* Level 1: *N* = 27; Level 2: *N* = 9. CBO = Community-Based Organization, ILS = Implementation Leadership Scale, ICS = Implementation Climate Scale, OSC = Organizational Social Context, OAE = *Outreach and Approach* subscale of ESMHC., which measures SBMH clinicians’ embeddedness. For discussion about 3-way interaction with embeddedness, see Additional File 1.

**Table 12:** Models for Implementation citizenship behaviors: Fixed Effects of Implementation Context Factors

implementation outcome	Research Questions	Model based on IVs	Fixed effect Coefficients (partial Cohen’s d in parentheses)				
			CBO	School	OAE	IOA (CBO × School)	CBO × School × OAE
Implementation citizenship behaviors	Main effects	ILS	0.511 (1.227)	0.267 (0.915)	-	-	-
		ICS	1.055 (2.323)	0.107 (0.133)	-	-	-
		OSC1: Proficiency	0.056 (0.547)	−0.018 (−0.242)	-	-	-
		OSC2 Stress	0.051 (0.765)	−0.037 (−0.732)	-	-	-
		OSC3 Functionality	−0.012 (−0.124)	0.088 (0.589)	-	-	-
	2-way interaction	ILS	0.571 (2.106)	0.123 (0.743)	-	0.35 (3.375)	-
		ICS	1.07 (4.281)	−0.004 (−0.004)	-	0.459 (1.71)	-
		OSC1: Proficiency	0.06 (0.575)	−0.021 (−0.287)	-	−0.001 (−0.579)	-
		OSC2 Stress	0.069 (1.246)	−0.064 (−1.129)	-	−0.003 (−0.758)	-
		OSC3 Functionality	−0.014 (−0.142)	0.092 (0.496)	-	0.001 (0.064)	-
	3-way interaction	ILS	0.46 (1.334)	0.1 (0.288)	0.12 (0.079)	-	−0.97 (−0.345)
		ICS	1.453 (2.28)	0.04 (0.071)	−0.128 (−0.12)	-	6.759 (1.106)
		OSC1: Proficiency	0.069 (0.622)	−0.031 (−0.4)	−1.207 (−0.461)	-	0.008 (0.079)
		OSC2 Stress	0.06 (0.616)	−0.055 (−0.516)	−0.988 (−0.263)	-	0.007 (0.206)
		OSC3 Functionality	0.003 (0.026)	0.089 (0.472)	−1.088 (−0.444)	-	0.053 (0.499)

*Note.* Level 1: *N* = 27; Level 2: *N* = 9. CBO = Community-Based Organization, ILS = Implementation Leadership Scale, ICS = Implementation Climate Scale, OSC = Organizational Social Context, OAE = *Outreach and Approach* subscale of ESMHC., which measures SBMH clinicians’ embeddedness. For discussion about 3-way interaction with embeddedness, see Additional File 1.

## Data Availability

The de-identified datasets can be requested from the authors.

## Abbreviations

CBO: Community-based organization
EBP: evidence-based practice
EPIS: Exploration, Preparation, Implementation, Sustainment
ICS: Implementation Climate Scale
ILS: Implementation leadership Scale
IOA: inter-organizational alignment
MLM: multilevel model
OSC: organizational social context
OIC: organizational implementation context
SBMH: school-based mental health
